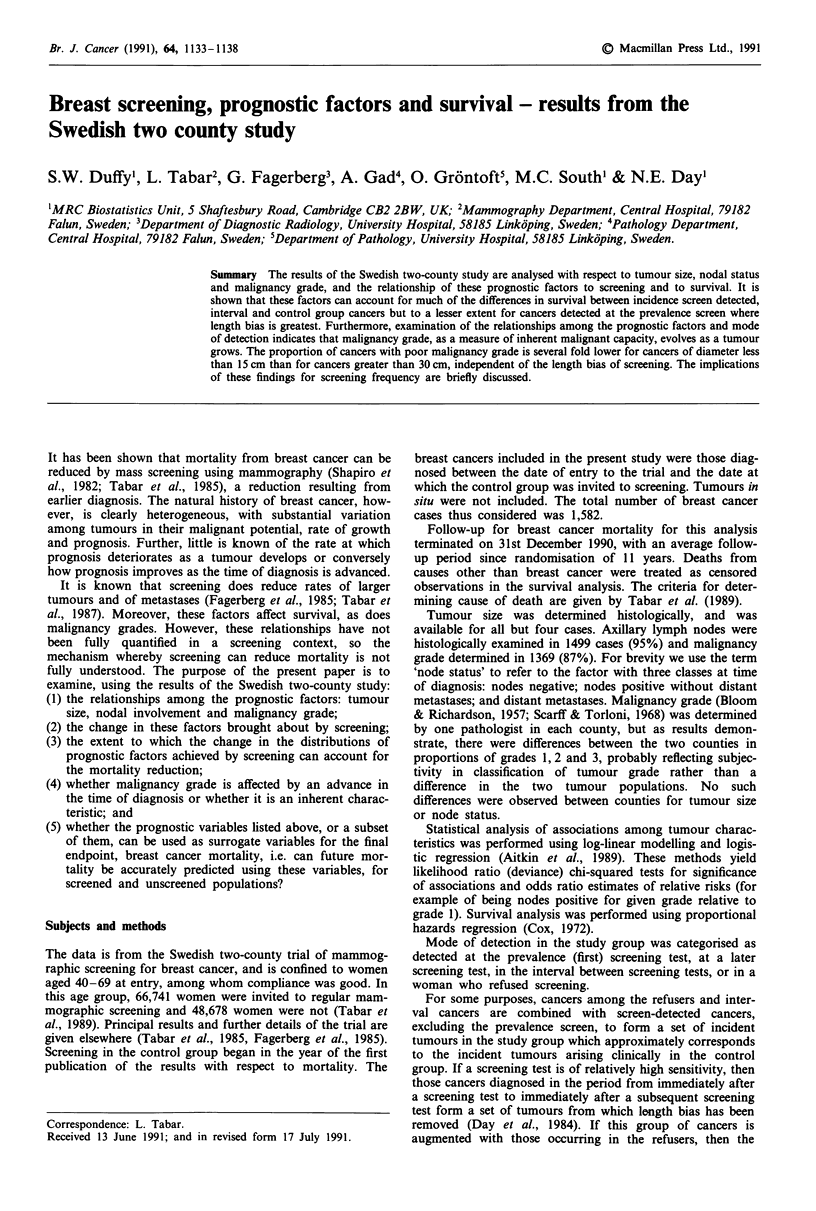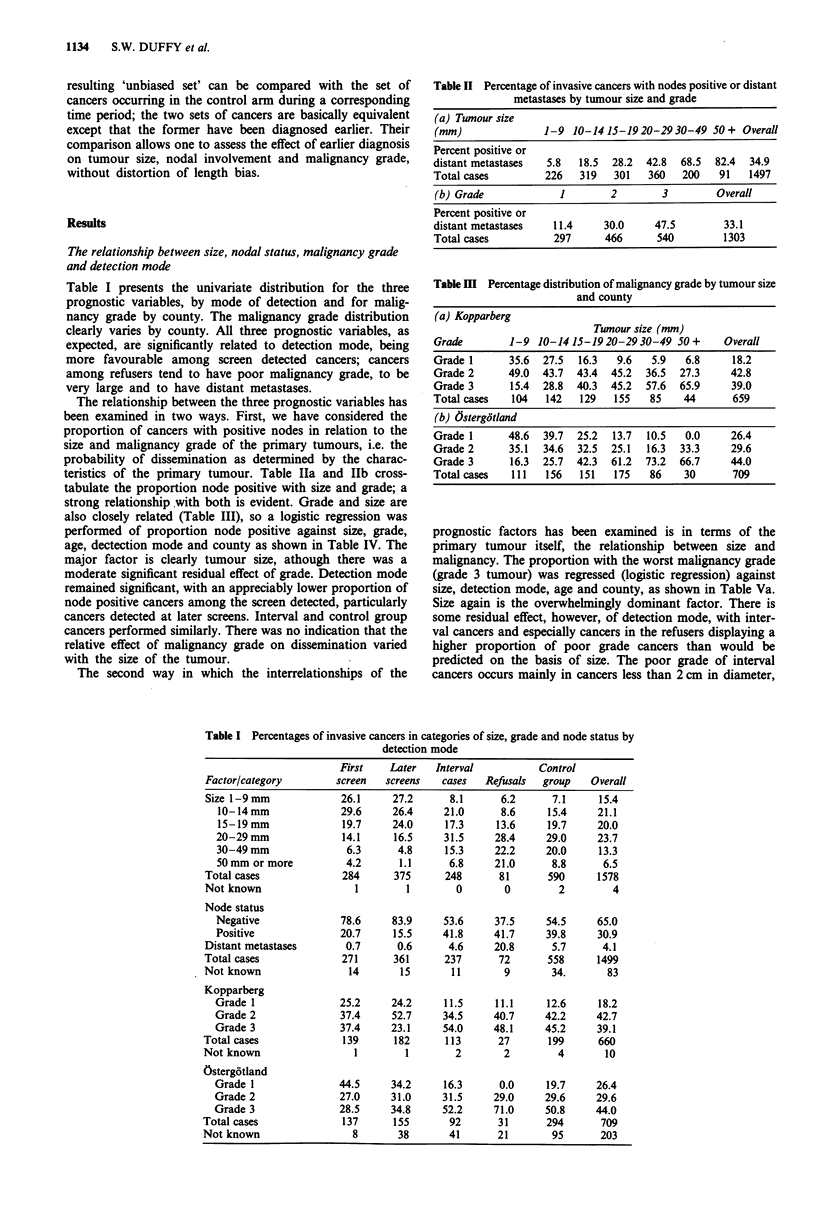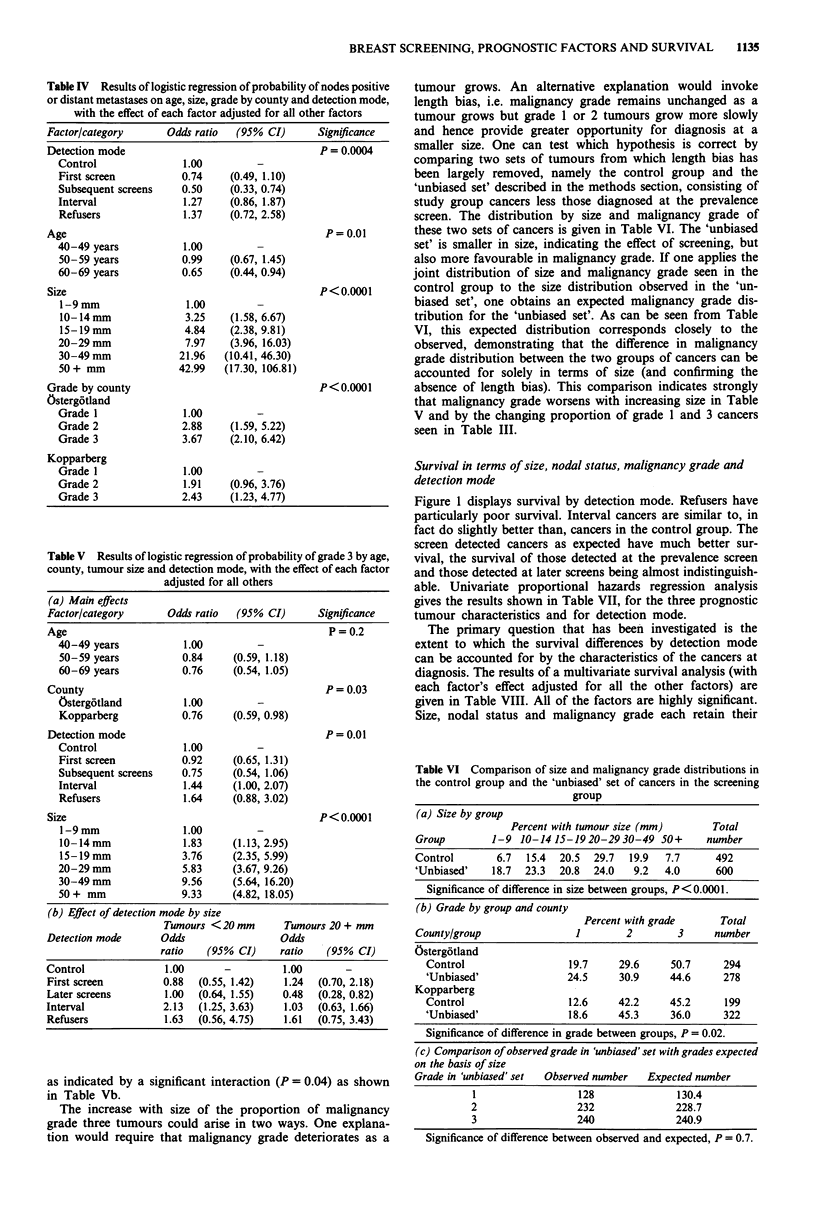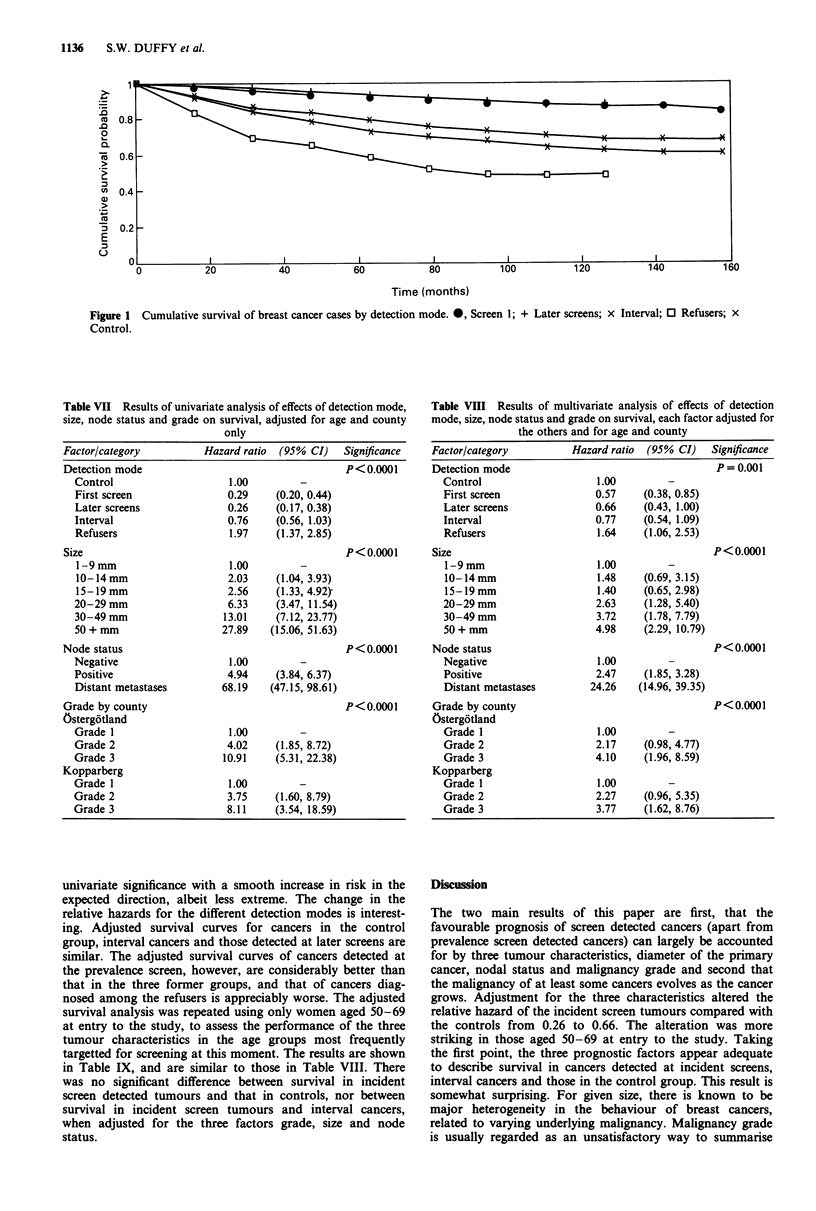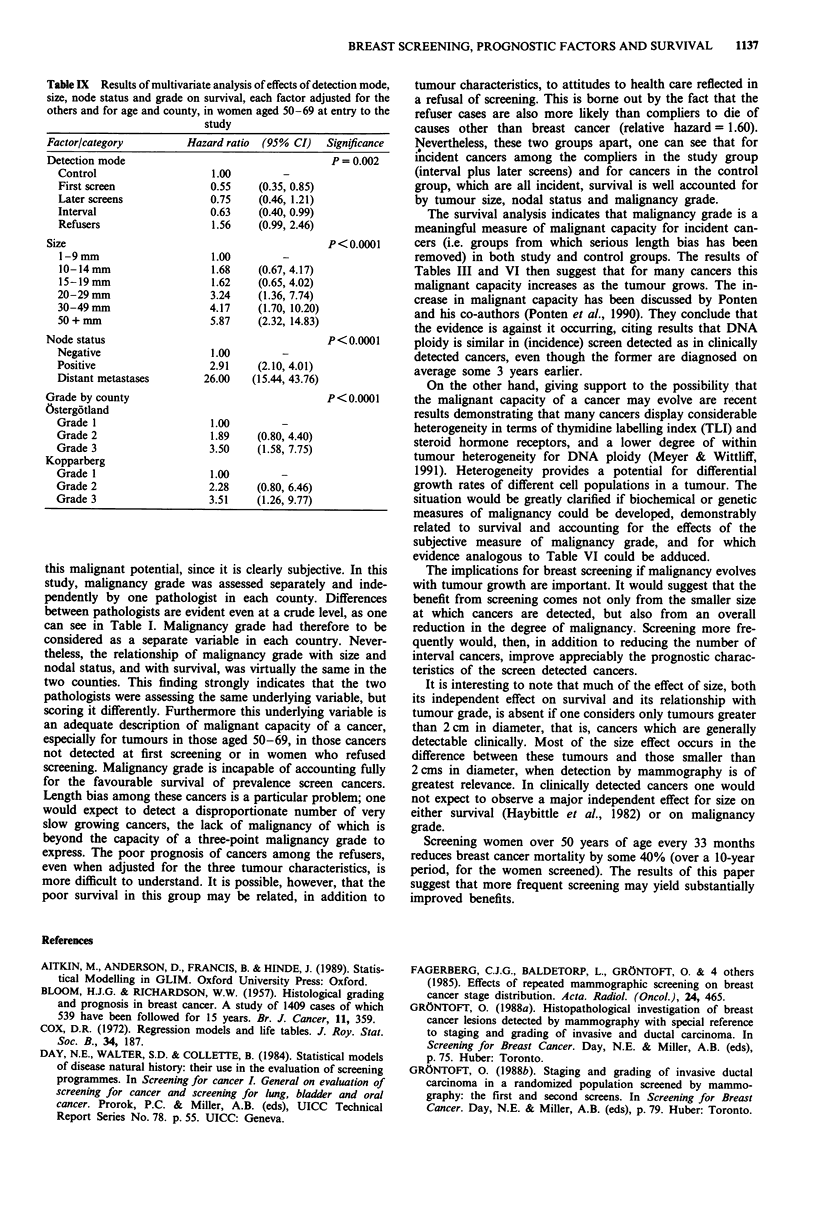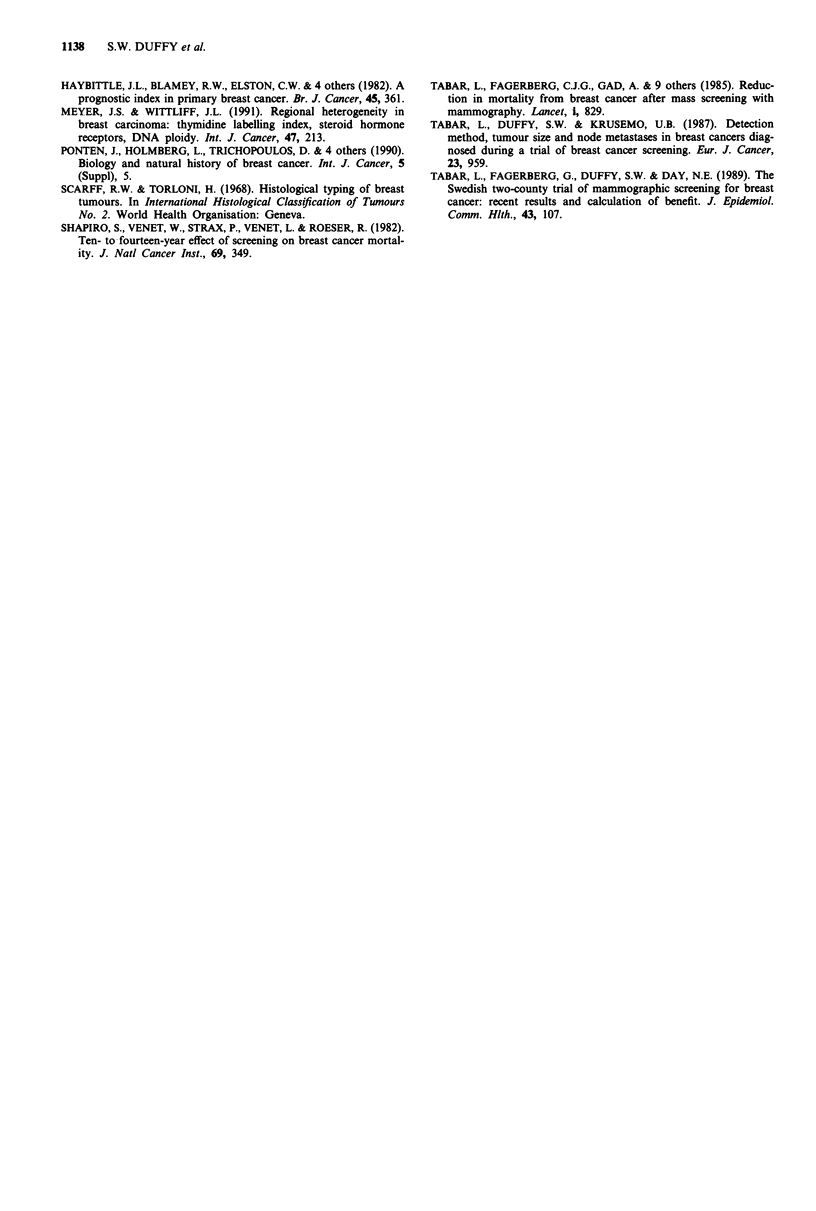# Breast screening, prognostic factors and survival--results from the Swedish two county study.

**DOI:** 10.1038/bjc.1991.477

**Published:** 1991-12

**Authors:** S. W. Duffy, L. Tabar, G. Fagerberg, A. Gad, O. Gröntoft, M. C. South, N. E. Day

**Affiliations:** MRC Biostatistics Unit, Cambridge, UK.

## Abstract

The results of the Swedish two-county study are analysed with respect to tumour size, nodal status and malignancy grade, and the relationship of these prognostic factors to screening and to survival. It is shown that these factors can account for much of the differences in survival between incidence screen detected, interval and control group cancers but to a lesser extent for cancers detected at the prevalence screen where length bias is greatest. Furthermore, examination of the relationships among the prognostic factors and mode of detection indicates that malignancy grade, as a measure of inherent malignant capacity, evolves as a tumour grows. The proportion of cancers with poor malignancy grade is several fold lower for cancers of diameter less than 15 cm than for cancers greater than 30 cm, independent of the length bias of screening. The implications of these findings for screening frequency are briefly discussed.


*Br. J. Cancer* (1991), **64**, 1133–1138 © Macmillan Press Ltd., 1991

### Breast screening, prognostic factors and survival – results from the Swedish two county study

S.W. Duffy[1], L. Tabar[2], G. Fagerberg[3], A. Gad[4], O. Gröntoft[5], M.C. South[1] & N.E. Day[1]

[1]*MRC Biostatistics Unit, 5 Shaftesbury Road, Cambridge CB2 2BW, UK;* [2]*Mammography Department, Central Hospital, 79182 Falun, Sweden;* [3]*Department of Diagnostic Radiology, University Hospital, 58185 Linköping, Sweden;* [4]*Pathology Department, Central Hospital, 79182 Falun, Sweden;* [5]*Department of Pathology, University Hospital, 58185 Linköping, Sweden.*

**Summary** The results of the Swedish two-county study are analysed with respect to tumour size, nodal status and malignancy grade, and the relationship of these prognostic factors to screening and to survival. It is shown that these factors can account for much of the differences in survival between incidence screen detected, interval and control group cancers but to a lesser extent for cancers detected at the prevalence screen where length bias is greatest. Furthermore, examination of the relationships among the prognostic factors and mode of detection indicates that malignancy grade, as a measure of inherent malignant capacity, evolves as a tumour grows. The proportion of cancers with poor malignancy grade is several fold lower for cancers of diameter less than 15 cm than for cancers greater than 30 cm, independent of the length bias of screening. The implications of these findings for screening frequency are briefly discussed.

It has been shown that mortality from breast cancer can be reduced by mass screening using mammography (Shapiro *et al.*, 1982; Tabar *et al.*, 1985), a reduction resulting from earlier diagnosis. The natural history of breast cancer, however, is clearly heterogeneous, with substantial variation among tumours in their malignant potential, rate of growth and prognosis. Further, little is known of the rate at which prognosis deteriorates as a tumour develops or conversely how prognosis improves as the time of diagnosis is advanced.

It is known that screening does reduce rates of larger tumours and of metastases (Fagerberg *et al.*, 1985; Tabar *et al.*, 1987). Moreover, these factors affect survival, as does malignancy grades. However, these relationships have not been fully quantified in a screening context, so the mechanism whereby screening can reduce mortality is not fully understood. The purpose of the present paper is to examine, using the results of the Swedish two-county study:

(1) the relationships among the prognostic factors: tumour size, nodal involvement and malignancy grade;
(2) the change in these factors brought about by screening;
(3) the extent to which the change in the distributions of prognostic factors achieved by screening can account for the mortality reduction;
(4) whether malignancy grade is affected by an advance in the time of diagnosis or whether it is an inherent characteristic; and
(5) whether the prognostic variables listed above, or a subset of them, can be used as surrogate variables for the final endpoint, breast cancer mortality, i.e. can future mortality be accurately predicted using these variables, for screened and unscreened populations?

### Subjects and methods

The data is from the Swedish two-county trial of mammographic screening for breast cancer, and is confined to women aged 40–69 at entry, among whom compliance was good. In this age group, 66,741 women were invited to regular mammographic screening and 48,678 women were not (Tabar *et al.*, 1989). Principal results and further details of the trial are given elsewhere (Tabar *et al.*, 1985, Fagerberg *et al.*, 1985). Screening in the control group began in the year of the first publication of the results with respect to mortality. The breast cancers included in the present study were those diagnosed between the date of entry to the trial and the date at which the control group was invited to screening. Tumours *in situ* were not included. The total number of breast cancer cases thus considered was 1,582.

Follow-up for breast cancer mortality for this analysis terminated on 31st December 1990, with an average follow-up period since randomisation of 11 years. Deaths from causes other than breast cancer were treated as censored observations in the survival analysis. The criteria for determining cause of death are given by Tabar *et al.* (1989).

Tumour size was determined histologically, and was available for all but four cases. Axillary lymph nodes were histologically examined in 1499 cases (95%) and malignancy grade determined in 1369 (87%). For brevity we use the term 'node status' to refer to the factor with three classes at time of diagnosis: nodes negative; nodes positive without distant metastases; and distant metastases. Malignancy grade (Bloom & Richardson, 1957; Scarff & Torloni, 1968) was determined by one pathologist in each county, but as results demonstrate, there were differences between the two counties in proportions of grades 1, 2 and 3, probably reflecting subjectivity in classification of tumour grade rather than a difference in the two tumour populations. No such differences were observed between counties for tumour size or node status.

Statistical analysis of associations among tumour characteristics was performed using log-linear modelling and logistic regression (Aitkin *et al.*, 1989). These methods yield likelihood ratio (deviance) chi-squared tests for significance of associations and odds ratio estimates of relative risks (for example of being nodes positive for given grade relative to grade 1). Survival analysis was performed using proportional hazards regression (Cox, 1972).

Mode of detection in the study group was categorised as detected at the prevalence (first) screening test, at a later screening test, in the interval between screening tests, or in a woman who refused screening.

For some purposes, cancers among the refusers and interval cancers are combined with screen-detected cancers, excluding the prevalence screen, to form a set of incident tumours in the study group which approximately corresponds to the incident tumours arising clinically in the control group. If a screening test is of relatively high sensitivity, then those cancers diagnosed in the period from immediately after a screening test to immediately after a subsequent screening test form a set of tumours from which length bias has been removed (Day *et al.*, 1984). If this group of cancers is augmented with those occurring in the refusers, then the

Correspondence: L. Tabar.
Received 13 June 1991; and in revised form 17 July 1991.

resulting 'unbiased set' can be compared with the set of cancers occurring in the control arm during a corresponding time period; the two sets of cancers are basically equivalent except that the former have been diagnosed earlier. Their comparison allows one to assess the effect of earlier diagnosis on tumour size, nodal involvement and malignancy grade, without distortion of length bias.

### Results

### *The relationship between size, nodal status, malignancy grade and detection mode*

Table I presents the univariate distribution for the three prognostic variables, by mode of detection and for malignancy grade by county. The malignancy grade distribution clearly varies by county. All three prognostic variables, as expected, are significantly related to detection mode, being more favourable among screen detected cancers; cancers among refusers tend to have poor malignancy grade, to be very large and to have distant metastases.

The relationship between the three prognostic variables has been examined in two ways. First, we have considered the proportion of cancers with positive nodes in relation to the size and malignancy grade of the primary tumours, i.e. the probability of dissemination as determined by the characteristics of the primary tumour. Table IIa and IIb cross-tabulate the proportion node positive with size and grade; a strong relationship with both is evident. Grade and size are also closely related (Table III), so a logistic regression was performed of proportion node positive against size, grade, age, dectection mode and county as shown in Table IV. The major factor is clearly tumour size, athough there was a moderate significant residual effect of grade. Detection mode remained significant, with an appreciably lower proportion of node positive cancers among the screen detected, particularly cancers detected at later screens. Interval and control group cancers performed similarly. There was no indication that the relative effect of malignancy grade on dissemination varied with the size of the tumour.

The second way in which the interrelationships of the prognostic factors has been examined is in terms of the primary tumour itself, the relationship between size and malignancy. The proportion with the worst malignancy grade (grade 3 tumour) was regressed (logistic regression) against size, detection mode, age and county, as shown in Table Va. Size again is the overwhelmingly dominant factor. There is some residual effect, however, of detection mode, with interval cancers and especially cancers in the refusers displaying a higher proportion of poor grade cancers than would be predicted on the basis of size. The poor grade of interval cancers occurs mainly in cancers less than 2 cm in diameter,

**Table II** Percentage of invasive cancers with nodes positive or distant metastases by tumour size and grade

| *(a) Tumour size (mm)* | *1–9* | *10–14* | *15–19* | *20–29* | *30–49* | *50+* | *Overall* |
|---|---|---|---|---|---|---|---|
| Percent positive or distant metastases | 5.8 | 18.5 | 28.2 | 42.8 | 68.5 | 82.4 | 34.9 |
| Total cases | 226 | 319 | 301 | 360 | 200 | 91 | 1497 |

| *(b) Grade* | *1* | *2* | *3* | *Overall* |
|---|---|---|---|---|
| Percent positive or distant metastases | 11.4 | 30.0 | 47.5 | 33.1 |
| Total cases | 297 | 466 | 540 | 1303 |

**Table III** Percentage distribution of malignancy grade by tumour size and county

| *Grade* | *1–9* | *10–14* | *15–19* | *20–29* | *30–49* | *50+* | *Overall* |
|---|---|---|---|---|---|---|---|
| *(a) Kopparberg* | | | | | | | |
| Grade 1 | 35.6 | 27.5 | 16.3 | 9.6 | 5.9 | 6.8 | 18.2 |
| Grade 2 | 49.0 | 43.7 | 43.4 | 45.2 | 36.5 | 27.3 | 42.8 |
| Grade 3 | 15.4 | 28.8 | 40.3 | 45.2 | 57.6 | 65.9 | 39.0 |
| Total cases | 104 | 142 | 129 | 155 | 85 | 44 | 659 |
| *(b) Östergötland* | | | | | | | |
| Grade 1 | 48.6 | 39.7 | 25.2 | 13.7 | 10.5 | 0.0 | 26.4 |
| Grade 2 | 35.1 | 34.6 | 32.5 | 25.1 | 16.3 | 33.3 | 29.6 |
| Grade 3 | 16.3 | 25.7 | 42.3 | 61.2 | 73.2 | 66.7 | 44.0 |
| Total cases | 111 | 156 | 151 | 175 | 86 | 30 | 709 |

(Tumour size (mm) spans columns 1–9 to 50+.)

**Table I** Percentages of invasive cancers in categories of size, grade and node status by detection mode

| *Factor/category* | *First screen* | *Later screens* | *Interval cases* | *Refusals* | *Control group* | *Overall* |
|---|---|---|---|---|---|---|
| Size 1–9 mm | 26.1 | 27.2 | 8.1 | 6.2 | 7.1 | 15.4 |
| 10–14 mm | 29.6 | 26.4 | 21.0 | 8.6 | 15.4 | 21.1 |
| 15–19 mm | 19.7 | 24.0 | 17.3 | 13.6 | 19.7 | 20.0 |
| 20–29 mm | 14.1 | 16.5 | 31.5 | 28.4 | 29.0 | 23.7 |
| 30–49 mm | 6.3 | 4.8 | 15.3 | 22.2 | 20.0 | 13.3 |
| 50 mm or more | 4.2 | 1.1 | 6.8 | 21.0 | 8.8 | 6.5 |
| Total cases | 284 | 375 | 248 | 81 | 590 | 1578 |
| Not known | 1 | 1 | 0 | 0 | 2 | 4 |
| Node status | | | | | | |
| Negative | 78.6 | 83.9 | 53.6 | 37.5 | 54.5 | 65.0 |
| Positive | 20.7 | 15.5 | 41.8 | 41.7 | 39.8 | 30.9 |
| Distant metastases | 0.7 | 0.6 | 4.6 | 20.8 | 5.7 | 4.1 |
| Total cases | 271 | 361 | 237 | 72 | 558 | 1499 |
| Not known | 14 | 15 | 11 | 9 | 34. | 83 |
| Kopparberg | | | | | | |
| Grade 1 | 25.2 | 24.2 | 11.5 | 11.1 | 12.6 | 18.2 |
| Grade 2 | 37.4 | 52.7 | 34.5 | 40.7 | 42.2 | 42.7 |
| Grade 3 | 37.4 | 23.1 | 54.0 | 48.1 | 45.2 | 39.1 |
| Total cases | 139 | 182 | 113 | 27 | 199 | 660 |
| Not known | 1 | 1 | 2 | 2 | 4 | 10 |
| Östergötland | | | | | | |
| Grade 1 | 44.5 | 34.2 | 16.3 | 0.0 | 19.7 | 26.4 |
| Grade 2 | 27.0 | 31.0 | 31.5 | 29.0 | 29.6 | 29.6 |
| Grade 3 | 28.5 | 34.8 | 52.2 | 71.0 | 50.8 | 44.0 |
| Total cases | 137 | 155 | 92 | 31 | 294 | 709 |
| Not known | 8 | 38 | 41 | 21 | 95 | 203 |

**Table IV** Results of logistic regression of probability of nodes positive or distant metastases on age, size, grade by county and detection mode, with the effect of each factor adjusted for all other factors

| *Factor/category* | *Odds ratio* | *(95% CI)* | *Significance* |
|---|---|---|---|
| Detection mode | | | $P = 0.0004$ |
| Control | 1.00 | – | |
| First screen | 0.74 | (0.49, 1.10) | |
| Subsequent screens | 0.50 | (0.33, 0.74) | |
| Interval | 1.27 | (0.86, 1.87) | |
| Refusers | 1.37 | (0.72, 2.58) | |
| Age | | | $P = 0.01$ |
| 40–49 years | 1.00 | – | |
| 50–59 years | 0.99 | (0.67, 1.45) | |
| 60–69 years | 0.65 | (0.44, 0.94) | |
| Size | | | $P < 0.0001$ |
| 1–9 mm | 1.00 | – | |
| 10–14 mm | 3.25 | (1.58, 6.67) | |
| 15–19 mm | 4.84 | (2.38, 9.81) | |
| 20–29 mm | 7.97 | (3.96, 16.03) | |
| 30–49 mm | 21.96 | (10.41, 46.30) | |
| 50 + mm | 42.99 | (17.30, 106.81) | |
| Grade by county | | | $P < 0.0001$ |
| Östergötland | | | |
| Grade 1 | 1.00 | – | |
| Grade 2 | 2.88 | (1.59, 5.22) | |
| Grade 3 | 3.67 | (2.10, 6.42) | |
| Kopparberg | | | |
| Grade 1 | 1.00 | – | |
| Grade 2 | 1.91 | (0.96, 3.76) | |
| Grade 3 | 2.43 | (1.23, 4.77) | |

**Table V** Results of logistic regression of probability of grade 3 by age, county, tumour size and detection mode, with the effect of each factor adjusted for all others

| *(a) Main effects Factor/category* | *Odds ratio* | *(95% CI)* | *Significance* |
|---|---|---|---|
| Age | | | P = 0.2 |
| 40–49 years | 1.00 | – | |
| 50–59 years | 0.84 | (0.59, 1.18) | |
| 60–69 years | 0.76 | (0.54, 1.05) | |
| County | | | $P = 0.03$ |
| Östergötland | 1.00 | – | |
| Kopparberg | 0.76 | (0.59, 0.98) | |
| Detection mode | | | $P = 0.01$ |
| Control | 1.00 | – | |
| First screen | 0.92 | (0.65, 1.31) | |
| Subsequent screens | 0.75 | (0.54, 1.06) | |
| Interval | 1.44 | (1.00, 2.07) | |
| Refusers | 1.64 | (0.88, 3.02) | |
| Size | | | $P < 0.0001$ |
| 1–9 mm | 1.00 | – | |
| 10–14 mm | 1.83 | (1.13, 2.95) | |
| 15–19 mm | 3.76 | (2.35, 5.99) | |
| 20–29 mm | 5.83 | (3.67, 9.26) | |
| 30–49 mm | 9.56 | (5.64, 16.20) | |
| 50 + mm | 9.33 | (4.82, 18.05) | |

*(b) Effect of detection mode by size*

| *Detection mode* | *Tumours <20 mm* Odds ratio | *(95% CI)* | *Tumours 20 + mm* Odds ratio | *(95% CI)* |
|---|---|---|---|---|
| Control | 1.00 | – | 1.00 | – |
| First screen | 0.88 | (0.55, 1.42) | 1.24 | (0.70, 2.18) |
| Later screens | 1.00 | (0.64, 1.55) | 0.48 | (0.28, 0.82) |
| Interval | 2.13 | (1.25, 3.63) | 1.03 | (0.63, 1.66) |
| Refusers | 1.63 | (0.56, 4.75) | 1.61 | (0.75, 3.43) |

as indicated by a significant interaction ($P = 0.04$) as shown in Table Vb.

The increase with size of the proportion of malignancy grade three tumours could arise in two ways. One explanation would require that malignancy grade deteriorates as a tumour grows. An alternative explanation would invoke length bias, i.e. malignancy grade remains unchanged as a tumour grows but grade 1 or 2 tumours grow more slowly and hence provide greater opportunity for diagnosis at a smaller size. One can test which hypothesis is correct by comparing two sets of tumours from which length bias has been largely removed, namely the control group and the 'unbiased set' described in the methods section, consisting of study group cancers less those diagnosed at the prevalence screen. The distribution by size and malignancy grade of these two sets of cancers is given in Table VI. The 'unbiased set' is smaller in size, indicating the effect of screening, but also more favourable in malignancy grade. If one applies the joint distribution of size and malignancy grade seen in the control group to the size distribution observed in the 'unbiased set', one obtains an expected malignancy grade distribution for the 'unbiased set'. As can be seen from Table VI, this expected distribution corresponds closely to the observed, demonstrating that the difference in malignancy grade distribution between the two groups of cancers can be accounted for solely in terms of size (and confirming the absence of length bias). This comparison indicates strongly that malignancy grade worsens with increasing size in Table V and by the changing proportion of grade 1 and 3 cancers seen in Table III.

### *Survival in terms of size, nodal status, malignancy grade and detection mode*

Figure 1 displays survival by detection mode. Refusers have particularly poor survival. Interval cancers are similar to, in fact do slightly better than, cancers in the control group. The screen detected cancers as expected have much better survival, the survival of those detected at the prevalence screen and those detected at later screens being almost indistinguishable. Univariate proportional hazards regression analysis gives the results shown in Table VII, for the three prognostic tumour characteristics and for detection mode.

The primary question that has been investigated is the extent to which the survival differences by detection mode can be accounted for by the characteristics of the cancers at diagnosis. The results of a multivariate survival analysis (with each factor's effect adjusted for all the other factors) are given in Table VIII. All of the factors are highly significant. Size, nodal status and malignancy grade each retain their

**Table VI** Comparison of size and malignancy grade distributions in the control group and the 'unbiased' set of cancers in the screening group

*(a) Size by group*

| *Group* | *Percent with tumour size (mm)* 1–9 | 10–14 | 15–19 | 20–29 | 30–49 | 50+ | *Total number* |
|---|---|---|---|---|---|---|---|
| Control | 6.7 | 15.4 | 20.5 | 29.7 | 19.9 | 7.7 | 492 |
| 'Unbiased' | 18.7 | 23.3 | 20.8 | 24.0 | 9.2 | 4.0 | 600 |

Significance of difference in size between groups, $P < 0.0001$.

*(b) Grade by group and county*

| *County/group* | *Percent with grade* 1 | 2 | 3 | *Total number* |
|---|---|---|---|---|
| Östergötland | | | | |
| Control | 19.7 | 29.6 | 50.7 | 294 |
| 'Unbiased' | 24.5 | 30.9 | 44.6 | 278 |
| Kopparberg | | | | |
| Control | 12.6 | 42.2 | 45.2 | 199 |
| 'Unbiased' | 18.6 | 45.3 | 36.0 | 322 |

Significance of difference in grade between groups, $P = 0.02$.

*(c) Comparison of observed grade in 'unbiased' set with grades expected on the basis of size*

| *Grade in 'unbiased' set* | *Observed number* | *Expected number* |
|---|---|---|
| 1 | 128 | 130.4 |
| 2 | 232 | 228.7 |
| 3 | 240 | 240.9 |

Significance of difference between observed and expected, $P = 0.7$.

**Figure 1** Cumulative survival of breast cancer cases by detection mode. ●, Screen 1; + Later screens; × Interval; □ Refusers; × Control.

**Table VII** Results of univariate analysis of effects of detection mode, size, node status and grade on survival, adjusted for age and county only

| *Factor/category* | *Hazard ratio* | *(95% CI)* | *Significance* |
|---|---|---|---|
| Detection mode | | | $P<0.0001$ |
| Control | 1.00 | – | |
| First screen | 0.29 | (0.20, 0.44) | |
| Later screens | 0.26 | (0.17, 0.38) | |
| Interval | 0.76 | (0.56, 1.03) | |
| Refusers | 1.97 | (1.37, 2.85) | |
| Size | | | $P<0.0001$ |
| 1–9 mm | 1.00 | – | |
| 10–14 mm | 2.03 | (1.04, 3.93) | |
| 15–19 mm | 2.56 | (1.33, 4.92) | |
| 20–29 mm | 6.33 | (3.47, 11.54) | |
| 30–49 mm | 13.01 | (7.12, 23.77) | |
| 50 + mm | 27.89 | (15.06, 51.63) | |
| Node status | | | $P<0.0001$ |
| Negative | 1.00 | – | |
| Positive | 4.94 | (3.84, 6.37) | |
| Distant metastases | 68.19 | (47.15, 98.61) | |
| Grade by county | | | $P<0.0001$ |
| Östergötland | | | |
| Grade 1 | 1.00 | – | |
| Grade 2 | 4.02 | (1.85, 8.72) | |
| Grade 3 | 10.91 | (5.31, 22.38) | |
| Kopparberg | | | |
| Grade 1 | 1.00 | – | |
| Grade 2 | 3.75 | (1.60, 8.79) | |
| Grade 3 | 8.11 | (3.54, 18.59) | |

**Table VIII** Results of multivariate analysis of effects of detection mode, size, node status and grade on survival, each factor adjusted for the others and for age and county

| *Factor/category* | *Hazard ratio* | *(95% CI)* | *Significance* |
|---|---|---|---|
| Detection mode | | | $P=0.001$ |
| Control | 1.00 | – | |
| First screen | 0.57 | (0.38, 0.85) | |
| Later screens | 0.66 | (0.43, 1.00) | |
| Interval | 0.77 | (0.54, 1.09) | |
| Refusers | 1.64 | (1.06, 2.53) | |
| Size | | | $P<0.0001$ |
| 1–9 mm | 1.00 | – | |
| 10–14 mm | 1.48 | (0.69, 3.15) | |
| 15–19 mm | 1.40 | (0.65, 2.98) | |
| 20–29 mm | 2.63 | (1.28, 5.40) | |
| 30–49 mm | 3.72 | (1.78, 7.79) | |
| 50 + mm | 4.98 | (2.29, 10.79) | |
| Node status | | | $P<0.0001$ |
| Negative | 1.00 | – | |
| Positive | 2.47 | (1.85, 3.28) | |
| Distant metastases | 24.26 | (14.96, 39.35) | |
| Grade by county | | | $P<0.0001$ |
| Östergötland | | | |
| Grade 1 | 1.00 | – | |
| Grade 2 | 2.17 | (0.98, 4.77) | |
| Grade 3 | 4.10 | (1.96, 8.59) | |
| Kopparberg | | | |
| Grade 1 | 1.00 | – | |
| Grade 2 | 2.27 | (0.96, 5.35) | |
| Grade 3 | 3.77 | (1.62, 8.76) | |

univariate significance with a smooth increase in risk in the expected direction, albeit less extreme. The change in the relative hazards for the different detection modes is interesting. Adjusted survival curves for cancers in the control group, interval cancers and those detected at later screens are similar. The adjusted survival curves of cancers detected at the prevalence screen, however, are considerably better than that in the three former groups, and that of cancers diagnosed among the refusers is appreciably worse. The adjusted survival analysis was repeated using only women aged 50–69 at entry to the study, to assess the performance of the three tumour characteristics in the age groups most frequently targetted for screening at this moment. The results are shown in Table IX, and are similar to those in Table VIII. There was no significant difference between survival in incident screen detected tumours and that in controls, nor between survival in incident screen tumours and interval cancers, when adjusted for the three factors grade, size and node status.

### Discussion

The two main results of this paper are first, that the favourable prognosis of screen detected cancers (apart from prevalence screen detected cancers) can largely be accounted for by three tumour characteristics, diameter of the primary cancer, nodal status and malignancy grade and second that the malignancy of at least some cancers evolves as the cancer grows. Adjustment for the three characteristics altered the relative hazard of the incident screen tumours compared with the controls from 0.26 to 0.66. The alteration was more striking in those aged 50–69 at entry to the study. Taking the first point, the three prognostic factors appear adequate to describe survival in cancers detected at incident screens, interval cancers and those in the control group. This result is somewhat surprising. For given size, there is known to be major heterogeneity in the behaviour of breast cancers, related to varying underlying malignancy. Malignancy grade is usually regarded as an unsatisfactory way to summarise

**Table IX** Results of multivariate analysis of effects of detection mode, size, node status and grade on survival, each factor adjusted for the others and for age and county, in women aged 50–69 at entry to the study

| *Factor/category* | *Hazard ratio* | *(95% CI)* | *Significance* |
|---|---|---|---|
| Detection mode | | | $P = 0.002$ |
| Control | 1.00 | – | |
| First screen | 0.55 | (0.35, 0.85) | |
| Later screens | 0.75 | (0.46, 1.21) | |
| Interval | 0.63 | (0.40, 0.99) | |
| Refusers | 1.56 | (0.99, 2.46) | |
| Size | | | $P < 0.0001$ |
| 1–9 mm | 1.00 | – | |
| 10–14 mm | 1.68 | (0.67, 4.17) | |
| 15–19 mm | 1.62 | (0.65, 4.02) | |
| 20–29 mm | 3.24 | (1.36, 7.74) | |
| 30–49 mm | 4.17 | (1.70, 10.20) | |
| 50 + mm | 5.87 | (2.32, 14.83) | |
| Node status | | | $P < 0.0001$ |
| Negative | 1.00 | – | |
| Positive | 2.91 | (2.10, 4.01) | |
| Distant metastases | 26.00 | (15.44, 43.76) | |
| Grade by county | | | $P < 0.0001$ |
| Östergötland | | | |
| Grade 1 | 1.00 | – | |
| Grade 2 | 1.89 | (0.80, 4.40) | |
| Grade 3 | 3.50 | (1.58, 7.75) | |
| Kopparberg | | | |
| Grade 1 | 1.00 | – | |
| Grade 2 | 2.28 | (0.80, 6.46) | |
| Grade 3 | 3.51 | (1.26, 9.77) | |

this malignant potential, since it is clearly subjective. In this study, malignancy grade was assessed separately and independently by one pathologist in each county. Differences between pathologists are evident even at a crude level, as one can see in Table I. Malignancy grade had therefore to be considered as a separate variable in each country. Nevertheless, the relationship of malignancy grade with size and nodal status, and with survival, was virtually the same in the two counties. This finding strongly indicates that the two pathologists were assessing the same underlying variable, but scoring it differently. Furthermore this underlying variable is an adequate description of malignant capacity of a cancer, especially for tumours in those aged 50–69, in those cancers not detected at first screening or in women who refused screening. Malignancy grade is incapable of accounting fully for the favourable survival of prevalence screen cancers. Length bias among these cancers is a particular problem; one would expect to detect a disproportionate number of very slow growing cancers, the lack of malignancy of which is beyond the capacity of a three-point malignancy grade to express. The poor prognosis of cancers among the refusers, even when adjusted for the three tumour characteristics, is more difficult to understand. It is possible, however, that the poor survival in this group may be related, in addition to tumour characteristics, to attitudes to health care reflected in a refusal of screening. This is borne out by the fact that the refuser cases are also more likely than compliers to die of causes other than breast cancer (relative hazard = 1.60). Nevertheless, these two groups apart, one can see that for incident cancers among the compliers in the study group (interval plus later screens) and for cancers in the control group, which are all incident, survival is well accounted for by tumour size, nodal status and malignancy grade.

The survival analysis indicates that malignancy grade is a meaningful measure of malignant capacity for incident cancers (i.e. groups from which serious length bias has been removed) in both study and control groups. The results of Tables III and VI then suggest that for many cancers this malignant capacity increases as the tumour grows. The increase in malignant capacity has been discussed by Ponten and his co-authors (Ponten *et al.*, 1990). They conclude that the evidence is against it occurring, citing results that DNA ploidy is similar in (incidence) screen detected as in clinically detected cancers, even though the former are diagnosed on average some 3 years earlier.

On the other hand, giving support to the possibility that the malignant capacity of a cancer may evolve are recent results demonstrating that many cancers display considerable heterogeneity in terms of thymidine labelling index (TLI) and steroid hormone receptors, and a lower degree of within tumour heterogeneity for DNA ploidy (Meyer & Wittliff, 1991). Heterogeneity provides a potential for differential growth rates of different cell populations in a tumour. The situation would be greatly clarified if biochemical or genetic measures of malignancy could be developed, demonstrably related to survival and accounting for the effects of the subjective measure of malignancy grade, and for which evidence analogous to Table VI could be adduced.

The implications for breast screening if malignancy evolves with tumour growth are important. It would suggest that the benefit from screening comes not only from the smaller size at which cancers are detected, but also from an overall reduction in the degree of malignancy. Screening more frequently would, then, in addition to reducing the number of interval cancers, improve appreciably the prognostic characteristics of the screen detected cancers.

It is interesting to note that much of the effect of size, both its independent effect on survival and its relationship with tumour grade, is absent if one considers only tumours greater than 2 cm in diameter, that is, cancers which are generally detectable clinically. Most of the size effect occurs in the difference between these tumours and those smaller than 2 cms in diameter, when detection by mammography is of greatest relevance. In clinically detected cancers one would not expect to observe a major independent effect for size on either survival (Haybittle *et al.*, 1982) or on malignancy grade.

Screening women over 50 years of age every 33 months reduces breast cancer mortality by some 40% (over a 10-year period, for the women screened). The results of this paper suggest that more frequent screening may yield substantially improved benefits.

### References

AITKIN, M., ANDERSON, D., FRANCIS, B. & HINDE, J. (1989). Statistical Modelling in GLIM. Oxford University Press: Oxford.

BLOOM, H.J.G. & RICHARDSON, W.W. (1957). Histological grading and prognosis in breast cancer. A study of 1409 cases of which 539 have been followed for 15 years. *Br. J. Cancer*, **11**, 359.

COX, D.R. (1972). Regression models and life tables. *J. Roy. Stat. Soc. B.*, **34**, 187.

DAY, N.E., WALTER, S.D. & COLLETTE, B. (1984). Statistical models of disease natural history: their use in the evaluation of screening programmes. In *Screening for cancer I. General on evaluation of screening for cancer and screening for lung, bladder and oral cancer*. Prorok, P.C. & Miller, A.B. (eds), UICC Technical Report Series No. 78. p. 55. UICC: Geneva.

FAGERBERG, C.J.G., BALDETORP, L., GRÖNTOFT, O. & 4 others (1985). Effects of repeated mammographic screening on breast cancer stage distribution. *Acta. Radiol. (Oncol.)*, **24**, 465.

GRÖNTOFT, O. (1988*a*). Histopathological investigation of breast cancer lesions detected by mammography with special reference to staging and grading of invasive and ductal carcinoma. In *Screening for Breast Cancer*. Day, N.E. & Miller, A.B. (eds), p. 75. Huber: Toronto.

GRÖNTOFT, O. (1988*b*). Staging and grading of invasive ductal carcinoma in a randomized population screened by mammography: the first and second screens. In *Screening for Breast Cancer*. Day, N.E. & Miller, A.B. (eds), p. 79. Huber: Toronto.

HAYBITTLE, J.L., BLAMEY, R.W., ELSTON, C.W. & 4 others (1982). A prognostic index in primary breast cancer. *Br. J. Cancer*, **45**, 361.

MEYER, J.S. & WITTLIFF, J.L. (1991). Regional heterogeneity in breast carcinoma: thymidine labelling index, steroid hormone receptors, DNA ploidy. *Int. J. Cancer*, **47**, 213.

PONTEN, J., HOLMBERG, L., TRICHOPOULOS, D. & 4 others (1990). Biology and natural history of breast cancer. *Int. J. Cancer*, **5** (Suppl), 5.

SCARFF, R.W. & TORLONI, H. (1968). Histological typing of breast tumours. In *International Histological Classification of Tumours No. 2.* World Health Organisation: Geneva.

SHAPIRO, S., VENET, W., STRAX, P., VENET, L. & ROESER, R. (1982). Ten- to fourteen-year effect of screening on breast cancer mortality. *J. Natl Cancer Inst.*, **69**, 349.

TABAR, L., FAGERBERG, C.J.G., GAD, A. & 9 others (1985). Reduction in mortality from breast cancer after mass screening with mammography. *Lancet*, **i**, 829.

TABAR, L., DUFFY, S.W. & KRUSEMO, U.B. (1987). Detection method, tumour size and node metastases in breast cancers diagnosed during a trial of breast cancer screening. *Eur. J. Cancer*, **23**, 959.

TABAR, L., FAGERBERG, G., DUFFY, S.W. & DAY, N.E. (1989). The Swedish two-county trial of mammographic screening for breast cancer: recent results and calculation of benefit. *J. Epidemiol. Comm. Hlth.*, **43**, 107.